# Crystal structure of a COG4313 outer membrane channel

**DOI:** 10.1038/srep11927

**Published:** 2015-07-07

**Authors:** Bert van den Berg, Satya Prathyusha Bhamidimarri, Mathias Winterhalter

**Affiliations:** 1 Institute for Cell and Molecular Biosciences, The Medical School, Newcastle University, Newcastle upon Tyne, NE2 4HH, UK; 2 School of Engineering and Science, Jacobs University Bremen, 28759 Bremen, Germany

## Abstract

COG4313 proteins form a large and widespread family of outer membrane channels and have been implicated in the uptake of a variety of hydrophobic molecules. Structure-function studies of this protein family have so far been hampered by a lack of structural information. Here we present the X-ray crystal structure of Pput2725 from the biodegrader *Pseudomonas putida* F1, a COG4313 channel of unknown function, using data to 2.3 Å resolution. The structure shows a 12-stranded barrel with an N-terminal segment preceding the first β-strand occluding the lumen of the barrel. Single channel electrophysiology and liposome swelling experiments suggest that while the narrow channel visible in the crystal structure does allow passage of ions and certain small molecules *in vitro*, Pput2725 is unlikely to function as a channel for hydrophilic molecules. Instead, the presence of bound detergent molecules inside the barrel suggests that Pput2725 mediates uptake of hydrophobic molecules. Sequence alignments and the locations of highly conserved residues suggest the presence of a dynamic lateral opening through which hydrophobic molecules might gain entry into the cell. Our results provide the basis for structure-function studies of COG4313 family members with known function, such as the SphA sphingosine uptake channel of *Pseudomonas aeruginosa*.

The outer membranes of Gram-negative bacteria contain many channels that mediate the diffusional uptake of nutrients and other small molecules required for cell growth and function^1^. Due to the presence of lipopolysaccharide on the cell surface, uptake channels are also required for hydrophobic molecules. For most outer membrane (OM) channel families, representative X-ray crystal structures of family members are known. One notable exception is the OM protein family known as Cluster of Orthologues Group (COG) 4313^2^. COG4313 proteins are also annotated as being involved in the meta-degradation pathway of phenol^3^. The UniProt database contains hundreds of family members, indicating that they are widespread in γ-proteobacteria. Although COG4313 family members vary widely in sequence, the mature proteins have similar lengths of 270−320 amino acid residues.

Only two COG4313 family members have been characterised in some detail in the literature. The first is the TcpY protein from *Cupriavidis necator* (formerly *Ralstonia eutropha*). This protein is located in the *tcp* operon which is dedicated to the degradation of trichlorophenol (TCP; Fig. 1A)^4^. Since TcpY has a signal sequence and is predicted to form a β-barrel structure, Belchik et al. suggested that TcpY could function as an OM uptake channel for TCP. To support this notion it was shown that *E. coli* transformed with plasmid-encoded *tcpY* is more susceptible to toxicity caused by TCP in the growth medium, supporting a role for TcpY as an uptake channel^4^. The second COG4313 protein for which a function has been proposed is the PA5325 gene from the opportunistic pathogen *Pseudomonas aeruginosa.* PA5325 is strongly upregulated in the presence of sphingosine by the sphingosine-responsive transcription factor *sphR* (PA5324), and was renamed *sphA*^5^. Interestingly, individual deletions of both *sphR* and *sphA* result in decreased survival of *P. aeruginosa* in the murine lung. Sphingosine (Fig. 1A) is a compound with known antimicrobial properties and is a component of lung surfactant. Intriguingly, a *sphA* deletion results in increased killing of *P. aeruginosa* in mice. These data are consistent with a role for SphA as an uptake channel for sphingosine to protect the cell from the harmful affects of this surfactant, possibly by metabolising it^5^. With regards to the channel studied here, encoded by orf Pput2725 from the biodegrader *Pseudomonas putida* F1 (PpF1), no function has yet been established. However, the neighboring gene Pput2724 as well as Pput2727-2729 code for enzymes with roles in the degradation of monoaromatic hydrocarbons (MAH), suggesting a possible role for the Pput2725 protein in OM MAH uptake. Pput2725 is the only COG4313 family member in PpF1.

The available evidence for the COG4313 family therefore suggests that they form channels for OM uptake of a wide variety of hydrophobic molecules (hydrophobics). To date, the only OM protein family with an established role in the uptake of hydrophobics is that of the FadL family^6^. The archetype of the family, *E. coli* FadL, functions as an uptake channel for long-chain fatty acids (LCFA)^7^. FadL mediates uptake via lateral diffusion, a mechanism via which the substrate diffuses laterally from the barrel lumen into the OM via an opening in the wall of the barrel. In this way the polar layer of the LPS, which forms the principal barrier for diffusion of hydrophobics into the OM, is bypassed^6^,^8^. While the lateral diffusion model is established for highly hydrophobic molecules such as LCFA, it is not yet clear how less hydrophobic compounds such as mono-aromatic hydrocarbons (MAH) pass through FadL orthologs such as the toluene channel TodX from *Pseudomonas putida* F1 (PpF1). At least for TodX, the presence of a narrow “conventional” channel through the lumen of the barrel hints at the possibility of a classical uptake mechanism^9^. However, TodX also has a lateral opening at the same position as LCFA channels, making lateral diffusion a possibility for MAH uptake as well^9^.

Here we report the first structure of a COG4313 family member. The 2.3 Å resolution X-ray crystal structure of Pput2725 from PpF1 shows a 12-stranded β-barrel that is constricted on the periplasmic side by the N-terminal ~14 residues, which fold into the lumen of the barrel. Liposome swelling experiments and single channel electrophysiology suggest that Pput2725 most likely does not form a channel for transport of hydrophilic molecules. This notion is supported by the presence of two detergent molecules that are bound inside the barrel. Sequence alignments and the locations of highly conserved residues suggest the presence of a lateral diffusion exit site in the wall of the barrel. We propose that substrate uptake by COG4313 family members most likely occurs via lateral diffusion into the OM; however, classical transport directly into the periplasmic space cannot yet be ruled out. Our structure now enables the testing of these possibilities via structure-function studies of COG4313 family members with known functions.

## Results

### COG4313 channels form 12-stranded barrels with an inserted N-terminus

**Figure 1:**
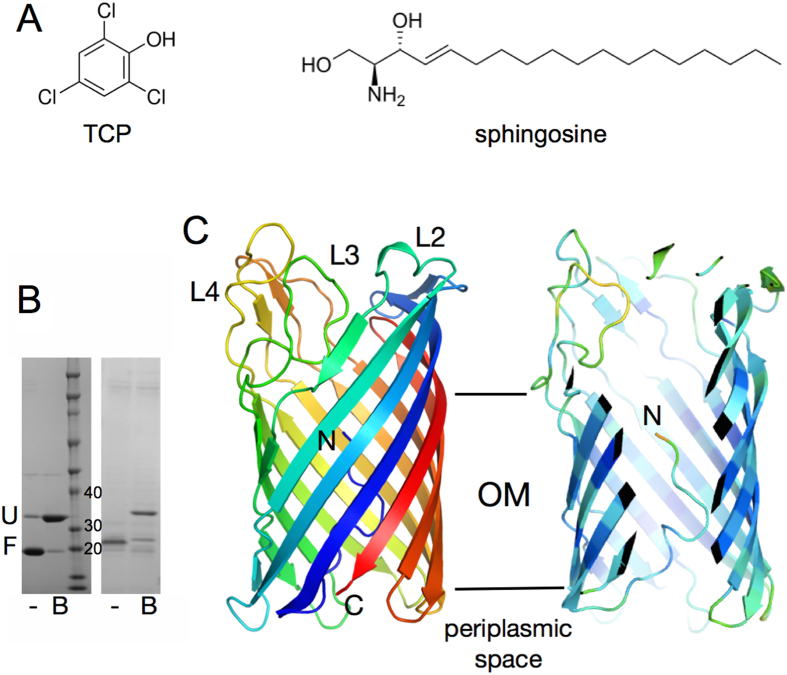
X-ray crystal structure of Pput2725. (**A**) Chemical structures of 2,4,6-trichlorophenol (TCP) and sphingosine, illustrating the diversity of COG4313 protein transport substrates. **(B**) Coomassie-stained SDS-PAGE gels showing *in vitro* folded Pput2725 (left panel) and OM-expressed CnTcpY (right panel). Both proteins are heat-modifiable and migrate at a lower molecular mass without sample boiling because they are still folded (**F**; -). After boiling (**B**) the proteins unfold (**U**) and migrate close to their true molecular mass (30.5 kDa for Pput2725 and 34 kDA for TcpY). (**C**) Cartoon overview of Pput2725 with rainbow coloring (N-terminus; blue). The right panel is a cut-away view showing the N-terminus inside the barrel lumen. The protein is colored by B-factor (blue; low). The extracellular loops and the N-terminus are labeled. All molecular models were made using Pymol^28^.

Initially we focused on the TcpY protein from *C. necator*, at the time the only COG4313 protein with an assigned function^4^. Expression levels for TcpY, both for OM-inserted protein as well as for inclusion bodies, were however too low to pursue structural studies. We then switched to the COG4313 protein Pput2725 from *P. putida* F1 (PpF1). PpF1 is of interest since it is a versatile biodegrader strain capable of assimilating various MAH compounds such as benzene and toluene. Moreover, we previously solved the structure of the PpF1 toluene uptake channel TodX^9^, which might be functionally related to COG4313 proteins. The yield of inclusion bodies for Pput2725 was sufficient for *in vitro* folding and purification. SDS-PAGE gels of purified Pput2725 show heat modifiability that is characteristic of stable β-barrel proteins (Fig. 1B). The difference in apparent molecular mass between the folded and unfolded protein (~5 kDa) is similar to that of *C. necator* TcpY expressed in the OM (Fig. 1B), indicating that Pput2725 is likely correctly folded.

Upon purification, a number of crystallization trials were set up using both commercial and in-house screens. After initial hit optimisation we obtained well-diffracting crystals for the wild type protein but were unable to solve the structure by molecular replacement using various search models with 12 β-strands, the number predicted for most COG4313 family members^10^. In addition, heavy atom searches also proved unsuccessful. We next produced SeMet substituted protein via *in vitro* folding using the same procedure as for the wild type protein (Methods). Only one of the various crystal forms obtained for the SeMet protein gave crystals with useable diffraction properties, and a highly redundant single anomalous dispersion (SAD) dataset to 2.8 Å was collected for the best crystal. The data were of sufficient quality for automated model building of a partial model that had two molecules in the asymmetric unit. Manual extension of the best-defined molecule resulted in a model that was successfully used for solving the high-resolution wild type Pput2725 structure at 2.3 Å by molecular replacement. The data collection and refinement statistics are summarised in Table 1.

**Figure 2:**
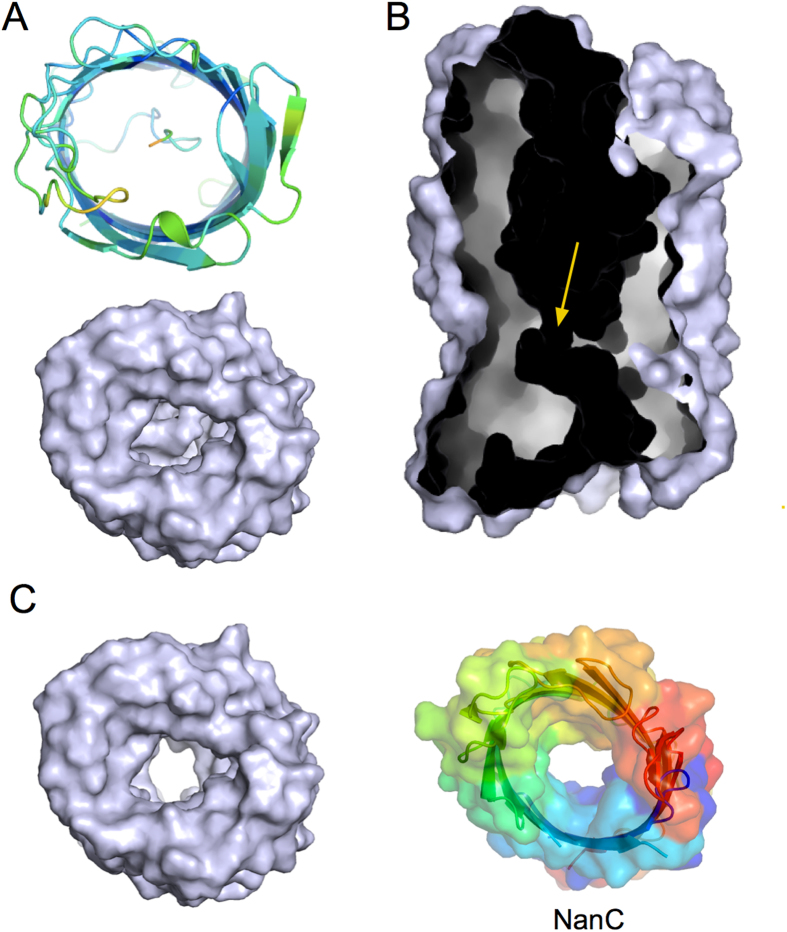
The N-terminus constricts the barrel lumen of Pput2725. (**A**) Views from the extracellular side showing a cartoon (top panel; colored by B-factor) and a molecular surface model (bottom panel). (**B**) Cut-away surface view showing the internal channel. The constriction is indicated with an arrow. (**C**) Surface view from the extracellular side of Pput2725 lacking the N-terminus, showing a large channel. For comparison, a rainbow surface model is shown for *E. coli* NanC, which is involved in the uptake of N-acetylneuraminic acid.

The crystal structure for the Pput2725 channel reveals a monomeric 12-stranded β-barrel, confirming the topology predictions for this family member and demonstrating that contrary to a previous notion made for TcpY^4^, COG4313 proteins do not have the same topology as FadL channels, which have 14-stranded barrels. Electron density for Pput2725 is visible for the entire polypeptide, with the exception of a number of exposed side chains that are presumably disordered. On the extracellular side, there are no loops that fold inwards to constrict the channel as observed for barrels with larger diameters. However, on the periplasmic side the N-terminal fourteen residues occupy the lumen of the barrel (Fig. 1C). This is a marked contrast with the structures of members of the KdgM family, which are so far the only other 12-stranded uptake channels for which structures have been solved^11^,^12^. For example, the structure of the *E. coli* N-Acetylneuraminic acid channel NanC shows a large channel of uniform diameter that is not constricted by the N-terminus (Fig. 2C). Thus, the barrel-inserted N-terminus of Pput2725 is a novel feature for 12-stranded OM uptake channels. The Pput2725 structure resembles that of the post-cleavage state of autotransporter proteins, which are not uptake channels for small molecules but protein secretion devices^13^. The structural similarity of Pput2725 to autotransporters is confirmed by a DALI search^14^, which shows that the most similar proteins to Pput2725 are all β-barrel domains of autotransporters, with the serine protease EspP being the top hit (PDB ID 3SLT; Z-score 22, 2.6 Å r.m.s.d.). It should be noted that while these proteins are similar in structure, the r.m.s.d. values are still relatively large and rationalise the failure of molecular replacement trials in solving the structure of Pput2725.

Despite the fact that the N-terminal domain of Pput2725 is relatively small it affects the architecture of the channel dramatically. Without the N-terminus, Pput2725 would have a large channel of ~10–12 Å in diameter (atom center to atom center), reminiscent of that in NanC (Fig. 2C). However, due to the presence of the N-terminus the lumen of the barrel is occluded almost completely, so that only a very narrow, convoluted channel is present that connects the extracellular milieu with the periplasmic space (Fig. 2A,B). This channel is lined with both hydrophobic and hydrophilic residues, and is constricted at the narrowest point by the side chains of residues Asp7, Tyr21 and Arg51. The extracellular cavity is strongly electronegative in character, which is somewhat surprising given the likely function of COG4313 proteins as uptake channels for hydrophobic molecules. To test whether Pput2725 could support the translocation of hydrophilic small molecules, possibly via the narrow observed “classical” channel, we conducted single channel electrophysiological experiments and liposome swelling assays.

### Pput2725 allows uptake of small polar molecules

**Figure 3:**
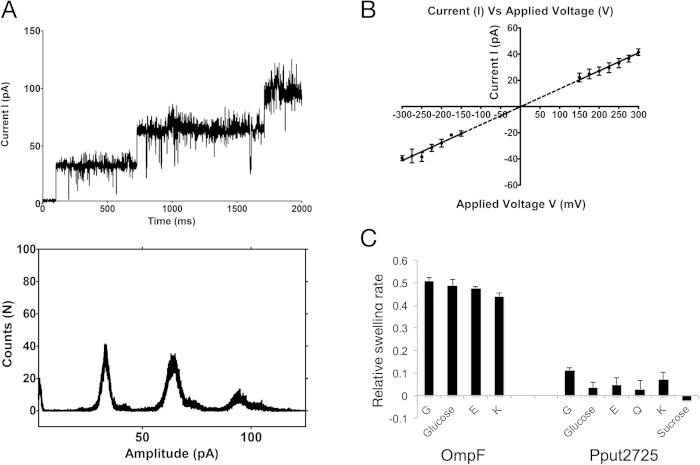
Ion currents and small molecule transport mediated by Pput2725. (**A**) Ion current trace of three subsequent single channel insertions into a solvent free membrane of DPhPC at +250 mV applied voltage, each giving rise to a single conductance state of ~30 pA. The lower panel shows the ion current amplitude histogram of this trace, indicating in addition to the zero conductive state a distribution around three distinctive current states. The conductance value of each channel is ~126 pS. Experimental conditions are 1M KCl 10 mM MES pH 6 and T = 20 °C. (**B**) Current (I) *vs.* Voltage (**V**) curve of Pput2725. The I–V curve is obtained from at least four independent measurements. (**C**) Relative substrate uptake rates shown as ΔOD_400_ values for the large-diameter OmpF porin from *E. coli* and for Pput2725. The following substrates were tested for Pput2725: glycine (**G**), glutamic acid (**E**), glutamine (**Q**), lysine (**K**), glucose and sucrose. Representative averages for 3–4 experiments are shown together with their standard deviations.

Upon reconstitution into a planar lipid bilayer, Pput2725 forms narrow channels with an average conductance of 126 pS in 1 M KCl 10 mM MES at pH6 (Fig. 3A). Surprisingly however, open channels are observed only at voltages higher than ±150 mV; at lower voltages the channels are predominantly in a non-conducting state. Given that any potential difference across the OM will be very small if at all present, the electrophysiology data suggest that Pput2725 may not form an ion-conducting channel under physiological conditions. The relatively low conductance values suggest that even at high voltages the N-terminus may be present inside the barrel lumen, as observed in the crystal structure. By comparison, the single-channel conductance observed for NanC in the presence of 1 M KCl (~350 pS at pH 7) is almost 3-fold higher than Pput2725, and the NanC traces are also quite stable^15^. The ion current traces for Pput2725 are very noisy and indicate a fluctuating behavior of the narrow channel that likely reflects movement of the N-terminus within the interior of the barrel. The channel is slightly more open in terms of conductance and duration of the open state at positive voltages compared to negative voltages. At high voltages the ionic current through the channel increases linearly with applied voltage (Fig. 3B). Together the electrophysiology data indicate the presence of narrow channel in Pput2725 that requires a substantial input of energy in order to open.

To test directly whether substrate translocation through Pput2725 occurs without an applied voltage we performed liposome swelling experiments^16^. For this, multi-lamellar phosphatidylcholine vesicles are loaded with a high molecular weight sugar that does not permeate the channel under investigation. The vesicles are then diluted into an iso-osmotic substrate solution. Channel-mediated substrate diffusion into the vesicles is accompanied by water uptake, causing the vesicles to swell and burst, allowing measurement of uptake rates by monitoring the decrease in OD_400_ of the vesicle suspension. For comparison of the uptake activities we also measured swelling rates for the nonspecific large-diameter OmpF porin^1^ from *E. coli*. As expected, OmpF mediates robust and similar uptake for all tested substrates, *i.e*. glycine, lysine, glutamate and glucose (Fig. 3C). In the case of Pput2725, we observed clear uptake activities only for the smallest substrate tested (glycine; 75 Da). For other substrates (glucose, lysine, glutamic acid and glutamine) the uptake activities are very low or non-significant, at best suggestive of slow permeation through a narrow pore (Fig. 3C). For larger substrates (sucrose; 340 Da) no uptake activity can be measured, consistent with the small-diameter channel formed by Pput2725. It should also be noted that due to the requirements of the assay, the employed substrate concentrations are quite high (~15 mM for most substrates). Taken together, our interpretation of these results is that Pput2725 may mediate the “leaking” of certain small hydrophilic molecules into the cell but that it is unlikely to function as an uptake channel for such compounds.

### Pput2725 binds detergent molecules

**Figure 4:**
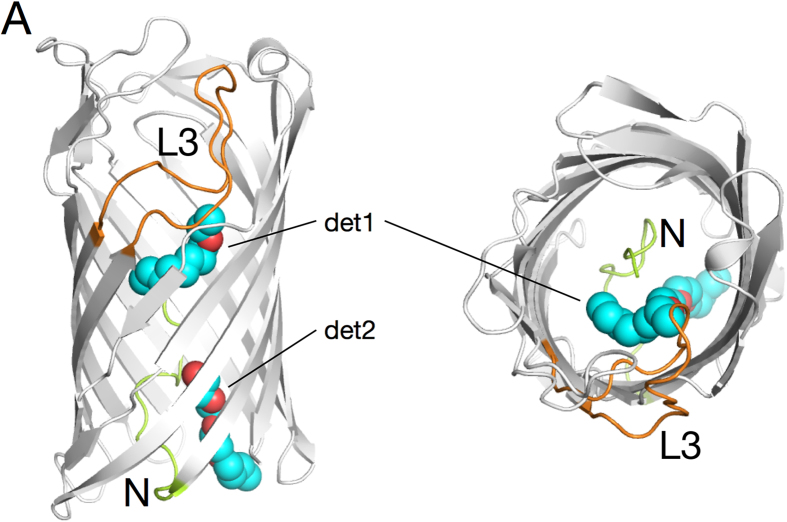
Detergent binding by Pput2725. Cartoon overviews of Pput2725 from the side and from the outside of the cell, showing the two C_8_E_4_ detergent molecules (det1 and det2) bound in the structure (carbons, cyan; oxygens, red). The N-terminus is colored green and loop L3 is shown in orange.

What then is the transport substrate for Pput2725? Since the function of this protein is not yet known, it is not possible to answer this question conclusively. However, the crystal structure does provide a clue as to the nature of the substrate. Two tubular stretches of density are present in the Pput2725 structure, and these likely correspond to partially ordered C_8_E_4_ detergent molecules used for crystallisation (Fig. 4). One of the detergent molecules (det1) is bound in the external vestibule, with the other (det2) bound on the periplasmic side below the constriction. Our results therefore suggest that the transport substrate of Pput2725 is amphipathic, analogous to that of TcpY and SphA.

### Sequence alignments of COG4313 proteins provide functional insights

**Figure 5:**
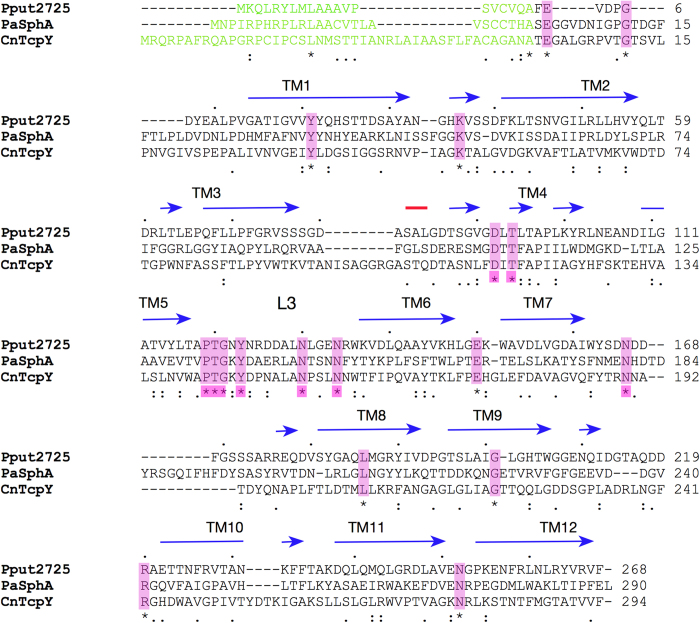
ClustalW sequence alignment of the COG4313 channels Pput2725, *C. necator* TcpY and *P. aeruginosa* SphA. The signal sequences as predicted by SignalP are shown in green. The observed secondary structure elements based on the crystal structure of Pput2725 are indicated (β-strands; blue, helices; red). Identical residues (*) located within the putative lateral exit site are highlighted in pink.

To obtain more information regarding function we performed a sequence alignment of Pput2725 with *C. necator* TcpY (CnTcpY) and *P. aeruginosa* SphA (PaSphA), the two COG4313 family members for which a function has been described. As is typical for the entire family, pairwise sequence identities between the three proteins are low (typically less than 20%), which should increase the information content of highly conserved residues. For the three COG4313 proteins analysed, there are only seventeen identical residues (Fig. 5). Two of these residues (Glu2 and Gly6) are located in the N-terminus. The side chain of Glu2 interacts with the side chain of Asn225 in the barrel wall, stabilising the N-terminus. Gly6 might be important for folding of the N-terminus in the confined lumen of the barrel. Interestingly, the alignment also suggests that the N-termini (*i.e*. the segments preceding the first β-strand) of *C. necator* TcpY and *P. aeruginosa* SphA are approximately twice the length compared to that of Pput2725 (Fig. 5). Assuming that these longer N-termini also occlude the lumen of the TcpY and SphA barrels this suggests that these proteins may not have the narrow channel that is present in Pput2725. This reinforces the notion from the transport experiments described above that COG4313 proteins likely do not function as classical uptake channels for hydrophilic molecules.

**Figure 6:**
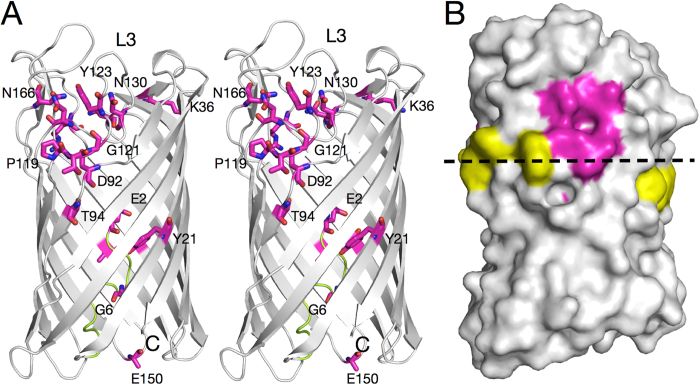
The location of conserved residues in Pput2725. (**A**) Stereo diagram viewed from the side, showing identical residues as magenta stick models (nitrogen atoms; blue, oxygen atoms; red). The N-terminus is colored green. (**B**) Surface model in a slightly different orientation relative to (**A**). Aromatic residues delineating the approximate external hydrophobic-hydrophilic interface of the OM (dashed line) are colored yellow.

When the remaining fifteen identical residues are highlighted onto the Pput2725 crystal structure, a remarkable pattern emerges. No fewer than nine residues in loops L2, L3 and L4 are clustered together (Asp92, Thr94, Pro119, Thr120, Gly121, Tyr123, Asn130, Asn134 and Asn166) (Fig. 6), hinting at a functional importance of this region. Most of the polar side chains appear to be involved in interactions stabilising the non-β-strand conformations of the loops. Viewed on a molecular surface model the conserved residues are clustered together and form a striking circular patch. The presence of the conserved cluster may have important implications for the possible transport mechanism, which will be discussed below.

## Discussion

We have solved the first structure of a COG4313 channel, providing the framework for a more detailed understanding of this protein family. The crystal structure of Pput2725 reveals a fold resembling that of the translocator domain of autotransporters, with a 12-stranded β-barrel that is partially occluded on the periplasmic side by the first ~14 residues of the N-terminus. A narrow channel is present in the crystal structure, connecting the extracellular environment with the periplasmic space. This channel is likely to be responsible for the uptake of ions and certain small hydrophilic compounds in the single channel and liposome swelling experiments (Fig. 3). However, the fact that the channel is closed at low voltages coupled to the low observed transport rates suggests that Pput2725 does not function as an uptake channel for hydrophilic compounds, in line with the consensus in the literature for other COG4313 proteins^3^,^4^,^5^.

**Figure 7:**
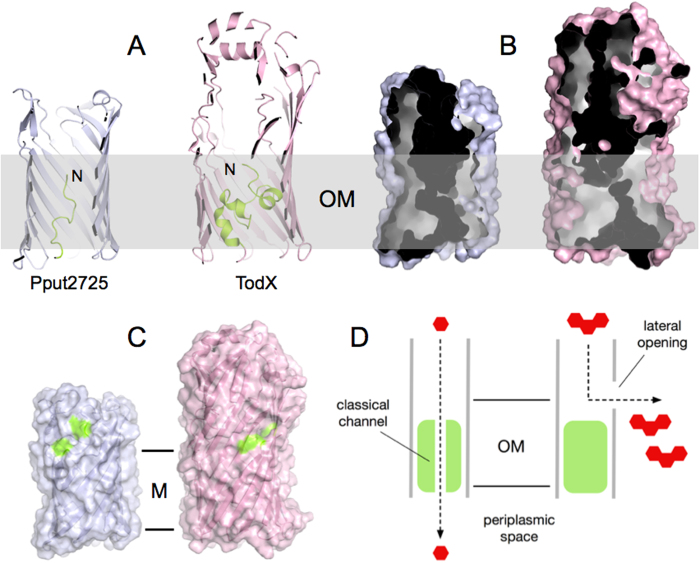
Comparison between Pput2725 and the toluene channel TodX. (**A**) Cut-away cartoon models viewed from the side, showing the barrel-occluding N-terminal domains. (**B**) Internal surfaces of the channels. The hydrophobic part of the OM is shown as a grey bar of ~25 Å wide (**C**). Surface views from the side, with residues in the Pput2725 lateral patch and on both sides of the TodX lateral opening colored green. The hydrophobic part of the OM (**M**) is delineated by horizontal lines. In all panels, Pput2725 and TodX are drawn to scale. (**D**) Possible models for substrate transport by COG4313 channels. Transport may occur either via a “classical” channel through the N-terminal domain into the periplasmic space (left), or via a lateral opening into the OM (right).

The likely non-polar nature of the transported substrates and the presence of an N-terminal domain occluding the barrel warrant a comparison of COG4313 proteins with members of the FadL family, in particular those involved in transport of mono-aromatic hydrocarbons (MAH) such as the PpF1 toluene channel TodX^9^. Due to the larger size of TodX (420 residues), its occluding N-terminal domain is considerably larger (~40 residues) compared to that in Pput2725 (Fig. 7A). Despite this difference both proteins may have a classical channel through the N-terminal domain (Fig. 7B), raising the possibility that moderately hydrophobic transport substrates gain access to the periplasmic space via this channel. The presence of a bound detergent molecule in the Pput2725 channel (Fig. 4; det2) provides some support for such a “classical” transport mechanism.

An alternative possibility for the transport mechanism in Pput2725 is lateral diffusion, which is emerging as a general mechanism for OM insertion and transport of hydrophobic and amphipathic molecules such as lipopolysaccharide (LPS) and long chain fatty acids (LCFA)^6^,^8^,^17^,^18^,^19^. Here the substrates diffuse from the lumen of the barrel, via a lateral opening in the barrel wall, into the OM. Energetically, lateral diffusion makes sense for hydrophobic/amphipathic molecules because the OM serves as an efficient sink for such compounds, enabling transport to be driven forward via mass action. However, there is no lateral opening in the barrel wall in the Pput2725 crystal structure. Instead, there is a cluster of highly conserved residues in the barrel wall (Fig. 6). Relative to the boundaries of the OM, the conserved cluster is located at the same position as the lateral opening in TodX (Fig. 7C) and *E. coli* FadL. We therefore hypothesise that the conserved cluster can generate a lateral opening, suggesting in turn that lateral diffusion operates as the transport mechanism (Fig. 7D). The interfacial location of the opening would allow the partitioning of hydrophobic and hydrophilic parts of amphipathic molecules upon their emergence from the lateral opening^6^,^8^. Given that the putative lateral opening in Pput2725 is closed, a conformational change, possibly resulting from substrate binding as in *E. coli* FadL^20^, would be required to generate the opening in the barrel wall. Interestingly, one of the bound detergent molecules (det1) is adjacent to the conserved lateral cluster (Fig. 4) and is in fact contacted by one of the conserved residues (Thr94), demonstrating that there is a binding site for hydrophobic molecules close to the putative exit site.

The fact that the transport substrate of Pput2725 is not known and the lack of a COG4313 family member in a tractable model system (*i.e*. *E. coli*) precludes the determination of a transport mechanism in our current study by structure-function experiments. However, our work provides a starting point for further structural and *in vivo* functional studies on COG4313 family members with an established function, such as *Pseudomonas aeruginosa* SphA. Such efforts should be focused on modifying the N-terminal region as well as the conserved lateral cluster to elucidate the transport mechanism by which COG4313 proteins move their substrates across the OM.

## Methods

### Cloning, protein expression and purification

For production of Pput2725 (UniProt ID: A5W3Z9) protein from inclusion bodies, the mature region of Pput2725 was predicted with SignalP 4.1, and amplified from *P. putida* F1 genomic DNA by PCR, with an ATG start codon added at the 5’ end of the gene and a sequence coding for a hexa-histidine tag added at the 3’ end. The amplicon was digested with EcoR1 and XbaI and ligated into EcoRI/XbaI digested pB22 vector. Protein expression was carried out in C43 (DE3) *E. coli* cells. For inclusion body production, cells were grown at 37 °C until OD600 ~ 0.6 and induced with 0.2% arabinose, followed by an additional 3 hrs growth at 37 °C. After harvesting by centrifugation, cells were ruptured by 1 pass through a cell disrupter (Avestin Emulsiflex C-3) operated at 20 kpsi. Inclusion bodies (IBs) were isolated by centrifugation for 10,000 rpm for 20 mins (45Ti rotor, Beckmann). IBs were resuspended at room temperature with 1% Triton X-100 in 10 mM Tris/50 mM NaCl pH 8 and centrifuged again. This wash step was repeated twice without added Triton X-100. The IBs were solubilized in 10 mM Tris/50 mM NaCl pH 8.0 containing 8 M urea by stirring overnight at room temperature, and centrifuged for 30 mins at 45,000 rpm to clarify the solution. For *in vitro* folding, the supernatant was diluted 10-fold by slow, dropwise addition to a stirred solution of 10 mM Tris/50 mM NaCl/1% decyl-β-maltoside (DM; Anatrace) and incubated overnight at 4 °C with slow stirring. Subsequently, NaCl was added to 250 mM and the folding mixture was loaded onto a 10 ml nickel affinity column (chelating sepharose, GE Biosciences). The column was washed with 150 ml TSB buffer (20 mM Tris/300 mM NaCl pH 8) containing 0.2% DM and 20 mM imidazole and eluted with 30 ml 250 mM imidazole. Monomeric Pput2725 was separated from aggregated material by gel filtration chromatography (Superdex-200) in 10 mM Hepes/100 mM NaCl/0.12% DM pH 7.5. For crystallization, a second gel filtration step was performed in 10 mM Hepes/100 mM NaCl/0.4% C_8_E_4_ pH 7.5. Protein fractions were pooled, concentrated to 10–15 mg/ml and aliquots were flash frozen in liquid nitrogen. Seleno-methionine (SeMet) substituted protein was produced by the methionine inhibition pathway in C43 cells in the same way as described above, using LeMasters/Richards minimal medium with 0.3% glycerol as carbon source. In this case, protein induction was carried out with 0.5% arabinose for 16 hrs at 37 °C. Two SeMet protein preparations in pure C_8_E_4_ buffer yielded only crystals that diffracted to low resolution. Those SeMet preparations were subsequently combined and ran on a Superdex-200 column in 10 mM Hepes/100 mM NaCl/0.3% C_8_E_4_/0.02% LDAO/0.05% octyl-β-glucoside. This detergent mixture did result in well-diffracting crystals.

The gene sequence of the mature region of *C. necator* TcpY was synthesized as a fusion with the *E. coli* OmpA signal sequence and a C-terminal hexa-histidine sequence for OM expression. The gene product was excised by digestion with EcoRI and XbaI, and ligated into EcoRI/XbaI digested pB22 vector. Production of OM-integrated *C. necator* TcpY was achieved by growing C43 Δ*cyoABCD* cells in LB medium till OD_600_ ~ 0.6, followed by induction with 0.1% arabinose for ~16 hrs at 20 °C. Cells were ruptured as described above, followed by ultracentrifugation at 45,000 rpm for 45 mins to collect total membranes. These were extracted with 2% (w/v) Elugent (Calbiochem) by stirring for 2 hrs at 4 °C followed by centrifugation at 42,000 rpm (30 mins). The clarified supernatant was loaded onto a nickel affinity column equilibrated in LDAO and washed and eluted as described above. Due to low yields no further purification was performed.

### Pput2725 crystallisation and structure determination

Both for wild type and SeMet substituted protein various commercial and in-house crystallization screens were set up using sitting drop vapour diffusion and Gryphon (Art Robbins Intruments) and Mosquito (TTP Labtech) crystallization robots. Well-diffracting wild type crystals were obtained from 20–25% PEG 4K, 0.05 M Hepes pH 7.5. The SeMet crystal used for structure solution grew from the MemGold2 screen (Molecular Dimensions), condition 1/42 (19% PEG 6K/0.05 M MOPS pH 7.0/0.2 M NaCl). Crystals were cryoprotected by short soaks (5–10 seconds) in mother liquor supplemented with 20% (v/v) glycerol. A highly redundant dataset (composed of 3 sweeps of 180 degree each) to 2.8 Å resolution was collected for one bar-shaped crystal. Data were processed using HKL2000^21^. Despite the suboptimal wavelength used for collection (0.92 Å, corresponding to a remote high-energy wavelength for selenium), eight heavy atom sites were found using HYSS within Phenix AUTOSOL^22^. Six of these are selenium sites (out of six expected for the two molecules within the asymmetric unit); the remaining 2 sites are close to histidine residues and might correspond to a divalent metal ion. Phenix AUTOBUILD^23^ generated a partial model that was extended manually within Coot^24^. When the R_free_ of the model reached ~35%, the most complete molecule was used for molecular replacement of the 2.3 Å resolution native dataset using Phaser. Several cycles of manual building and refinement within Phenix produced a model with reasonable statistics (Table 1). For the later stages in refinement, no non-crystallographic symmetry (NCS) restraints were used. In addition, isotropic individual B-factor refinement was used throughout (without TLS). The four molecules in the asymmetric unit are identical within error; molecule C has however the lowest B-factors and will therefore be discussed in this paper.

### Liposome swelling experiments

Liposome swelling experiments were performed according to a protocol originally described by Nikaido^25^ with minor modifications. Briefly, 100 mg egg-PC (25 mg/ml in chloroform; Avanti Polar Lipids) was combined with 1 ml chloroform containing 2 mg dicetylphosphate. 0.25 ml of this mixture (5 mg egg-PC) was evaporated under air and dried for 3 hrs under vacuum. The dried lipid film was resuspended in 0.3 ml water and divided into 100 μl aliquots. Each aliquot received 0.6 nmoles of protein purified in C_8_E_4_. Care was taken not to add more than 3 μl of detergent-purified protein to avoid liposome rupture. The control liposomes received the same amount of C_8_E_4_ buffer as the proteoliposomes. Subsequently the proteoliposomes were sonicated in a water bath for 1 min, followed by drying under vacuum overnight. Proteoliposomes were gently resuspended in 12 mM stachyose in 10 mM Hepes buffer pH 7.0. Measurements were started after 2 hrs incubation at room temperature. For each measurement, 3–10 μl of liposomes were added to 100 μl of substrate solution in 10 mM Hepes pH 7.0 followed by manual mixing. The empirically determined (iso-osmotic) substrate concentration (typically ~10–15 mM) did not change the OD_400_ of control liposomes by more than 2% during a 1 min time interval. Proteoliposome rupture due to substrate/water transport was monitored by following the decrease in OD_400_ for 1 min with 5 s intervals. The first 20 s of the curves were fitted to a straight line, with the slope taken as the swelling rate. The average (n = 3 or 4) swelling rates of the control liposomes was subtracted from each of the proteoliposome data curves.

### Single channel electrophysiology

Single channel recordings were performed as mentioned elsewhere^26^. Briefly, COG4313 Pput2725 channels were reconstituted in a solvent free bilayer using Montal and Muller technique^27^. Two symmetric home-made Teflon cuvette sandwiches were used with a 25 μm thick Teflon film having a round aperture of approximately 50–100 μm in diameter. The aperture was pre-painted with 1% Hexadecane in Hexane to render the pore aperture more lipophilic. After 30 min of drying the cuvettes were filled with buffer containing 1 M KCl. For bilayer formation a lipid monolayer was spread from 1% lipid solution in pentane. In our case we used Diphytanoylphosphatidylcholine, (DPhPC, Avanti Polar Lipids, Alabaster, AL). Ag/AgCl electrodes (World Precision Instruments, Sarasota, FL) were used to measure the electronic current. One electrode was connected to the ground (Cis side of the membrane). The other was connected to the headstage of an Axopatch 200B amplifier (Axon Instruments), also termed as the trans (live) electrode. Conductance measurements were performed in the Voltage Clamp mode of the Axopatch 200B amplifier and digitized by an Axon Digidata 1440A digitizer controlled by Clampex software (Axon Instruments, Foster city, CA). The current trace was filtered by low pass Bessel filter at 10 kHz, recorded onto a computer hard drive with a sampling frequency of 50 kHz. Data were analysed by the Clampfit program. Due to the low conductance of the channel and very noisy behavior, the traces were filtered at 1 kHz for obtaining ion current traces.

## Additional Information

**How to cite this article**: Van den Berg, B. *et al.* Crystal structure of a COG4313 outer membrane channel. *Sci. Rep.* **5**, 11927; doi: 10.1038/srep11927 (2015).

**Accession codes**: atomic coordinates and structure factors of Pput2725 have been deposited in the Protein Data Bank (http://wwpdb.org) with accession code 4RL8

## Figures and Tables

**Table 1:** Data collection and refinement statistics for Pput2725.

	SeMet	Wild type
Data collection		
Beamline	DLS i04-1	NSLS X25
Wavelength (Å)	0.92	1.1
Space group	P2_1_2_1_2_1_	P2_1_2_1_2_1_
Cell dimensions (a,b,c)	69.1, 74.4, 194.4	101.1, 120.3, 131.4
α,β,γ	90, 90, 90	90, 90, 90
Solvent content (%)	70	62
molecules/AU	2	4
Resolution (Å)	49–3.0	50–2.3
I/σI	29.5 (6.6)	9.0 (2.8)
Completeness (%)	99 (95)	100 (100)
Redundancy	19.7 (19.6)	9.2 (9.3)
Rmerge (%)	7.8 (48)	13.9 (66)
Rpim (%)	1.8 (11)	4.8 (23)
CC_1/2_	1.00 (0.99)	1.00 (0.92)
Phasing		
Sites found	8	
AutoSol FOM (score)	0.24 (31)	
Refinement		
Resolution (Å)		20–2.3
Unique reflections (n)		71454
R_work_/R_free_ (%)^a^		21.5 (25.3)
Atoms (n)		
protein/solvent/		8110/400
ligand/detergent		5/308
B factors (Å^2^)		
protein/solvent/		27/34
ligand/detergent		48/40
Rmsd		
bond lengths (Å)		0.006
bond angles (°)		1.01
Ramachandran plot (%)		
most favored/disallowed		98.9/0.0
Molprobity clashscore		8.0

Values in parentheses refer to the highest resolution shell.

^a^*R*_free_ was computed as for *R*_work_ using a test set (~5%) of randomly selected reflections that were omitted from the refinement.
